# Grandiose narcissism associates with higher cognitive performance under stress through more efficient attention distribution: An eye-tracking study

**DOI:** 10.1371/journal.pone.0302644

**Published:** 2024-05-03

**Authors:** Vasilena Stefanova, Christoph Scheepers, Paul Wilson, Kostas A. Papageorgiou

**Affiliations:** 1 College of Psychology, Birmingham City University, Birmingham, United Kingdom; 2 School of Psychology and Neuroscience, University of Glasgow, Glasgow, United Kingdom; 3 School of Psychology, Queen’s University Belfast, Belfast, United Kingdom; 4 Department of Psychology, Neapolis University Pafos, Pafos, Cyprus; University of Leeds, UNITED KINGDOM

## Abstract

Narcissism is a part of the Dark Triad that consists also of the traits of Machiavellianism and psychopathy. Two main types of narcissism exist: grandiose and vulnerable narcissism. Being a Dark Triad trait, narcissism is typically associated with negative outcomes. However, recent research suggests that at least the grandiose type may be linked (directly or indirectly) to positive outcomes including lower levels of psychopathology, higher school grades in adolescents, deeper and more strategic learning in university students and higher cognitive performance in experimental settings. The current pre-registered, quasi-experimental study implemented eye-tracking to assess whether grandiose narcissism indirectly predicts cognitive performance through wider distribution of attention on the Raven’s Progressive Matrices task. Fifty-four adults completed measures of the Dark Triad, self-esteem and psychopathology. Eight months to one year later, participants completed the Raven’s, while their eye-movements were monitored during high stress conditions. When controlling for previous levels of psychopathology, grandiose narcissism predicted higher Raven’s scores indirectly, through increased variability in the number of fixations across trials. These findings suggest that grandiose narcissism predicts higher cognitive performance, at least in experimental settings, and call for further research to understand the implications of this seemingly dark trait for performance across various settings.

## Introduction

The Dark Triad refers to the three interrelated traits of Machiavellianism, narcissism, and psychopathy [1]. The three traits share a dark core, that is, diminished empathy, ruthless exploitation of others [2], and a predisposition to high antagonism [3]. The present study focuses on the Dark Triad, and grandiose narcissism in particular, and their association with visual attention and intelligence.

Subclinical narcissism includes facets retained from the clinical syndrome, namely grandiosity, entitlement, dominance, and superiority. Two main types exist: grandiose and vulnerable narcissism. Exhibitionism, lack of humility/modesty, and interpersonal dominance characterize grandiose narcissism, whereas negative affect, distrust, selfishness, and a need for attention and recognition are key features of vulnerable narcissism [4]. Evidence is accumulating to suggest that grandiose and vulnerable narcissism form two separate factors within the Dark Triad with grandiose narcissism to not fit the Dark Triad core of callousness and manipulation [3]. Furthermore, and unlike vulnerable narcissism, grandiose narcissism has been shown to associate mostly—directly, indirectly or via its interaction with other traits—with positive outcomes such as fewer symptoms of psychopathology [5–8], higher income [9], more strategic learning in university students [10], higher mental toughness [11], higher self-rated creativity [12] and higher emotional intelligence [13] to name a few. Subclinical narcissism is associated with lower levels of depression and anxiety, suggesting that narcissism may offer a protective mechanism against psychopathology [6]. However, despite the plethora of evidence on the association between grandiose narcissism, and dark traits more generally, with diverse outcomes (e.g., psychopathology, other personality traits, occupational performance etc.) very few studies have explored their possible links with cognitive traits such as fluid intelligence.

## Intelligence and the Dark Triad

In a meta-analysis carried by O’Boyle and colleagues [14] no evidence was found to support a relationship between the Dark Triad and intelligence. A more recent meta-analysis by Stanek and Ones provided evidence of a link between Machiavellianism and fluid intelligence [15]. Another study by Kowalski and colleagues [16] reported that the tactical and analytical skills associated with Machiavellianism serve as an explanation of the trait’s positive association with fluid intelligence. The same study reported no associations among narcissism, psychopathy and fluid intelligence. Other studies reported a negative association between intelligence and psychopathy [17]. Narcissism has not been reliably linked with intelligence, neither negatively nor positively. However, some recent studies have shown positive indirect associations between grandiose narcissism and outcomes that link to higher intelligence. For example, one study reported an indirect association between grandiose narcissism and school grades [18]. More recently, grandiose narcissism was linked to enhanced cognitive performance on an arithmetic test and reduced stress [19]. This is in line with previous research, which showed that the performance of narcissists could in fact improve in adverse scenarios by encouraging hostile goal pursuit and a greater flexibility, as well as the use of compensatory behavioral strategies [20,21].

## Personality, intelligence, and eye-movements

Regardless of the breadth of studies tapping into different dimensions of dark traits and intelligence, the psychometric approach falls short in explaining the mechanisms underlying such relationships [22]. Additionally, it presents a series of biases hindering the validity of the results obtained. On one hand, personality measures are affected by desirability biases [23], acquiescence [24] or faking [25]. On the other hand, personality and testing variables may also influence performance and intelligence scores [26]. As such, there is a pressing need for a multi-method approach to shed light on the association between personality and intelligence. A recent interest in the field of microbehaviors has emerged to fit that purpose [27,28]. Personality and cognition, both having a neurobiological basis, are likely to manifest through behaviors that “are potentially available to careful observers using normal sensory processes” [27].

Largely ignored in traditional literature, visual attention measured through eye-tracking technology, is a promising tool for conveying information about cognitive and attentional processes related to intelligence and personality traits [29]. With advances in eye-tracking technology, there has been an increased interest in using eye movement analysis to explore the association between eye movements and the allocation of visual and cognitive resources. For example, in a study conducted by Vigneau and colleagues, [30], university students were monitored during 14 selected items of the Raven’s Advanced Progressive Matrices test. The study reported that proportional time on the test item, latency to first alternation, and time distribution on the problem matrix were positively correlated to test scores, whereas the number of alternations between matrix and response choice and the gaze time spent on answer alternatives were negatively correlated to the test scores [30,31]. Similar findings were reported by Hayes and collagues, [32] in a study with 35 university students, which reported that a significant percentage of the variance on the participants’ scores on a Raven’s Advanced Progressive Matrices test was explained by eye-fixation patterns, where systematic scanning of the problem matrices and less toggling between matrix and responses were indicative of better performance. Relatedly, Laurence and colleagues [33] investigated the association between eye movement patterns and IQ test performance in a study with 34 participants who completed a digitalized version of the Wiener Matrizen-Test 2. The authors reported that participants who scored higher on the test showed less gaze transitions between the relevant areas of interest and the response alternatives [31,33]. The results indicate that eye movements can serve as a means to uncover mechanisms underlying cognitive processes.

The literature around eye-movement and personality expanded since Rauthmann and colleagues [29] first investigated the Big Five with eye-tracking. By using abstract stimuli, they found that those with higher level of neuroticism had longer dwelling time at stimuli in order to evaluate potential risks. In turn, those high in extraversion presented shorter dwelling time and a higher number of fixations matching the fast-paced behavior of extraverts. The main finding was that high neuroticism affected attentional control (i.e., the lack of selective attention, showing longer fixation duration and fewer fixations). In other words, individuals high in neuroticism may have difficulties in disengaging from non-relevant stimuli. However, no attempts were made to estimate the impact of these associations for cognitive performance. Taken together, evidence may suggest that the same pattern would be expected if studying intelligence. That is, personality traits that predict higher stress responses would not only account for lower intelligence scores through test anxiety, but it would also affect fluid intelligence through attention. We focus here on fluid intelligence, given that basic physiological measures will accurately represent innate problem-solving abilities, rather than acquired knowledge or crystallised intelligence [33].

A number of recent studies have used eye-tracking measures to study intelligence and cognitive skills [30–33]. Eye-tracking provides insight into how individuals process visual stimuli and adapt their cognitive strategies to achieve certain goals [34]. The direction of eye-movements are influenced by cognitive activity, and as such, can inform about underlying cognitive processes [35]. A review by Eckstein and colleagues [36] reported that eye tracking measures are very effective in providing information about one’s attention control and the cognitive strategies employed in complex tasks. More recently, a study by Tsukahara and colleagues [37] identified a positive correlation between baseline pupil size and fluid intelligence. Gaze patterns during an intelligence test have also been found to explain a substantial amount of variance in intelligence test performance [38]. These findings highlight the link between eye-movements and cognitive ability.

There are currently no studies that have formally addressed whether personality traits associate with cognitive performance through their influence on attentional processes. Grandiose narcissism is of particular interest here due to the aforementioned positive associations with outcomes that are most commonly influenced by intelligence such as higher school grades; and due to the previously reported [39] negative connections with neuroticism, a trait that has been associated with problems to disengage attention. Eye fixations are a measure widely used as a function of attention distribution [40]. During an eye fixation, the target object is positioned into the fovea and the point of gaze is stationary, allowing for visual input to be obtained [41]. In the current work, we used a range of eye-tracking measures as proxies for visual attention, in order to gain insight into the cognitive and attentional processes linked to intelligence and personality.

## The current study

The present repeated-measures study explored the indirect association of grandiose narcissism to cognitive performance (assessed using the Raven’s Progressive Matrices [42]) in stressful conditions. Elevated stress levels can hinder performance on tasks that require problem-solving, information recall and attention [43]. Therefore, it is important to study factors that boost performance under stress or offer a protective mechanism against it. Previous research has identified that narcissism is associated with improved resilience against stress, also resulting in improved cognitive performance [19]. The current work brings this all together through using a state-of-the-art eye-tracking setup to assess the distribution of visual attention during completion of the Raven’s test. Subsequently, we explored direct associations between grandiose narcissism and visual attention; grandiose narcissism and Raven’s score; and visual attention and Raven’s score. Previous research has demonstrated that flexibility in cognitive strategies employed during a problem-solving task predicts improved performance in the task [44]. In the current work, we aimed to explore whether narcissism may be linked to an improved performance in a problem-solving task through a more adaptive visual attention distribution in stress-inducing situations. Fixation duration refers to the time between saccadic eye movements when the eyes are relatively stable; it has been proposed that fixation duration could be a stable measure of individual differences in attention across both short and long test-retest intervals [45] and across different tasks [46,47]. Importantly for the current study, individual differences in fixation duration have been found to reliably correlate with intelligence [48]. As such, we decided to focus on fixation duration over any other eye-tracking measure. Considering that mean fixation duration could be made from a small number of long fixations or a large number of short fixations, we decided to focus on variation in number of fixations as a proxy measure of attention distribution. To clarify further, we were expecting that for participants that were highly stressed variation in number of fixations will be low contributing to poorer attention distribution at task, which in turn could lead to lower performance. Because we had evidence to suggest that narcissism predicts better responses under stress, we hypothesised that narcissists will exhibit better attention distribution as measured by variation in number of fixations achieving higher scores on the Raven’s test. We controlled for anxiety, depression and stress in the current analyses–as mentioned above, narcissism is linked to resilience against psychiatric symptoms [6].

We hypothesised that: (1) Similarly to other personality traits, grandiose narcissism will not predict Raven’s scores directly. Instead, (2) grandiose narcissism will associate positively with greater variability in visual attention across trials during stressful conditions; (3) Greater variability of visual attention that indicates more flexible and efficient processing of the stimuli will associate positively with Raven’s scores; (4) Grandiose narcissism will predict positively higher Raven’s scores, indirectly, through its positive association with visual attention that predicts higher Raven’s score.

This project was pre-registered on the Open Science Framework (OSF) on 08.07.2020 (https://doi.org/10.17605/OSF.IO/A37VH).

## Methods

### Participants

Τhe sample consisted of 54 participants (45 females and 9 males; *mean age =* 21.04; *SD =* 3.41), who were recruited from a pool of participants that had already provided self-report data on personality and psychopathology. Self-report data collection was performed from October 1^st^, 2018, to April 30^th^, 2019. Participants had given consent form to indicate their willingness to be contacted again to participate in any follow-up studies. Eye-tracking and cognitive ability data collection was performed from 19^th^ December 2019 to 30^th^ March 2020.

### Procedure

The Ethics Committee of the Faculty for Engineering and Physical Science at Queen’s University Belfast approved the study. Personality data (see Measures) were collected as part of a previous study approximately 1 year to 8 months before the start of the eye tracking study. Participants confirmed that they are available to take part in follow-up experiments at the time of completion of the personality assessments. Participants who previously completed the personality questionnaires were recruited to take part in the current follow-up study. Written consent forms were obtained twice for both the personality data collection and the experimental procedure. Participants were asked to complete the self-esteem measure [49] and the pre-task measure of acute stress [50]. Afterwards, they completed the Raven’s Progressive Matrices (RPM) [42] to assess general cognitive ability and during the task their eye movements were recorded using eye-tracking technology (SR-Research Eyelink 1000 Plus eye-tracker). The participants completed the task on a high-definition LCD with a screen resolution of 1280x1024 pixels. The sampling rate was 1000 Hz and the participants were positioned at a distance of approximately 70cm from the screen. Participants were randomly assigned to one of two conditions–high stress or low stress (see supplementary material I in S1 File).

After the task, participants were asked to complete the post-task measure of acute stress. After the data were collected, a preliminary analysis was performed to see if the manipulation worked as intended, which involved comparing the acute stress scores (measured using the Acute Stress Appraisals (ASA) scale which divides the stress induced by a task into pre- and post-task stress) of participants who were assigned to the high stress condition to those who were assigned to the low stress condition. Preliminary analyses (see supplementary material I in S1 File) uncovered two important findings: Going through the task was stressful for the participants; however, participants were not getting more stressed in the high stress condition as compared to the low stress condition. As such, data from the two conditions were collapsed into a single data set for all further analyses. This resulted in a deviation from our original hypotheses stated in the pre-registration protocol, specifically in the testing hypothesis two (introduced as “hypothesis three” in the pre-registration), which was tested based on the assumption that all participants engaged in a single, stressful condition (Table 3 below shows that all participants indicated increased levels of stress post-task than pre-task, suggesting that they found the task stress-inducing overall). The personality measures and visual attention measures are presented in Tables 1 and 2 below.

**Table 1:** Personality measures, psychopathology, stress, self-esteem and cognitive ability.

Variables	Name of measure	Reference
***Narcissism*, *Psychopathy and Machiavellianism***	***The Short Dark Triad*** ***questionnaire (SD3)***	Jones & Paulhus [51]
***Depression*, *Anxiety Depression*, *Anxiety and*** ***and Stress***	***Depression*, *Anxiety and Stress scale (DASS21)***	Lovibond & Lovibond [52]
***IQ score (general*** ***cognitive ability)***	***Raven’s Progressive Matrices (RPM)***	Raven et al. [42]
***Perceived stress pre- and post-task***	***Acute Stress Appraisals scale (ASA)***	Mendes et al. [50]
***Self-esteem***	***Rosenberg Self-esteem scale***	Rosenberg [49]

Note: For a detailed description of the measures refer to supplemental material I in S1 File.

**Table 2:** Visual attention measures. These variables were extracted via Trial Reports in SR-Research Data Viewer.

Name of measure	Description	Mean (M) across participants
***Overall number of fixations***	the sum of all eye fixations across trials	2136.24
***SD number of fixations***	the amount of variation in the overall number of fixations across trials	26.73
***Average fixation duration***	the average time for fixations during the task across all trials. Measures in milliseconds (ms)	226.53
***SD fixation duration***	the amount of variation in the duration of fixations across trials	38.43
***Overall number of saccades***	the sum of saccades across all trials	2092.93
***SD number of saccades***	the amount of variation in the overall number of saccades across trials	26.70
***Average saccade amplitude***	the mean distance of saccade travel	4.64
***SD saccade amplitude***	the amount of variation in the amplitude of saccades across trials	1.26
***Average saccade duration***	the time taken on average to complete a saccade. Measured in milliseconds (ms)	58.85
***SD saccade duration***	the amount of variation in the duration of saccades across trials	109.14
***Average reaction time***	the mean reaction time of participants when responding to test stimuli across trials. Measured in milliseconds (ms)	10276.41
***SD reaction time***	the amount of variation in reaction times across trials	8297.25
***Overall number of blinks***	the sum of blinks across all trials	132.39
***SD number of blinks***	the amount of variation in the number of blinks across trials	2.61
***Average blink duration***	represents the mean duration of blinks across all trials. Measured in milliseconds (ms)	195.66
***SD blink duration***	the amount of variation in the duration of blinks across trials	302.30

### Measures

#### Analysis plan

To test our first hypothesis, we explored whether narcissism predicts Raven’s score directly. We also tested hypothesis four, to explore whether narcissism predicts Raven’s score indirectly through SD number of fixations as a function of visual attention. As discussed earlier, we view SD number of fixations across trials as a proxy for strategic flexibility in solving the Raven’s items. A mediation analysis was conducted using Process MACRO [53], aiming to determine whether SD number of fixations mediates the effect of narcissism on Raven’s score. Anxiety, stress and depression were also included in the models as co-variates.

Secondly, we tested hypothesis two and investigated whether narcissism predicts visual attention measures. A multivariate multiple regression was conducted with narcissism as predictor and average fixation duration, SD fixation duration, overall number of fixations, SD number of fixations, average saccade amplitude, SD saccade amplitude, average saccade duration, SD saccade duration, overall number of saccades and SD number of saccades as outcome variables. Anxiety, depression and stress were included as co-variates.

Thirdly, we tested hypothesis three and assessed whether visual attention measures predict Raven’s score using linear regression. Average fixation duration, SD fixation duration, overall number of fixations, SD number of fixations, average saccade amplitude, SD saccade amplitude, average saccade duration, SD saccade duration, overall number of saccades and SD number of saccades were the predictors variables and Raven’s score was the outcome variable.

Finally, additional analyses identical to the ones conducted for narcissism were conducted with Machiavellianism, psychopathy and self-esteem as direct or indirect predictors of Raven’s score. (see supplementary material III in S1 File). Anxiety, stress and depression were added as co-variates. The aim of these additional “control” analyses is to explore whether the studied effects are caused by narcissism specifically or whether they can be observed for other Dark Triad traits or self-esteem.

Considering that personality has a stable component across the life span, both at the trait level and at the profile level [54], the predictor variables (personality) were assessed approximately two years prior to the collection of the eye-tracking data to make the results of the mediation analyses more reliable.

## Results

### Descriptive statistics

Descriptive statistics including means and standard deviations for the main and supplementary study variables are presented in Table 3.

**Table 3:** Descriptive statistics.

Variable	Mean (M)	Standard deviation (SD)	Cronbach’s alpha (α)
***Narcissism***	2.58	.55	.65
***Psychopathy***	1.87	.56	.75
***Machiavellianism***	2.90	.49	.64
***Depression***	1.74	.77	.89
***Anxiety***	1.86	.60	.75
***Stress***	2.14	.66	.80
***Raven’s score***	.69	.11	.85
***Pre-task stress***	-1.12	1.44	.47
***Self-esteem***	2.78	.40	.84
***Post-task stress***	.50	1.51	.45

### Testing the direct and indirect effect of narcissism on Raven’s score

**Fig 1:**
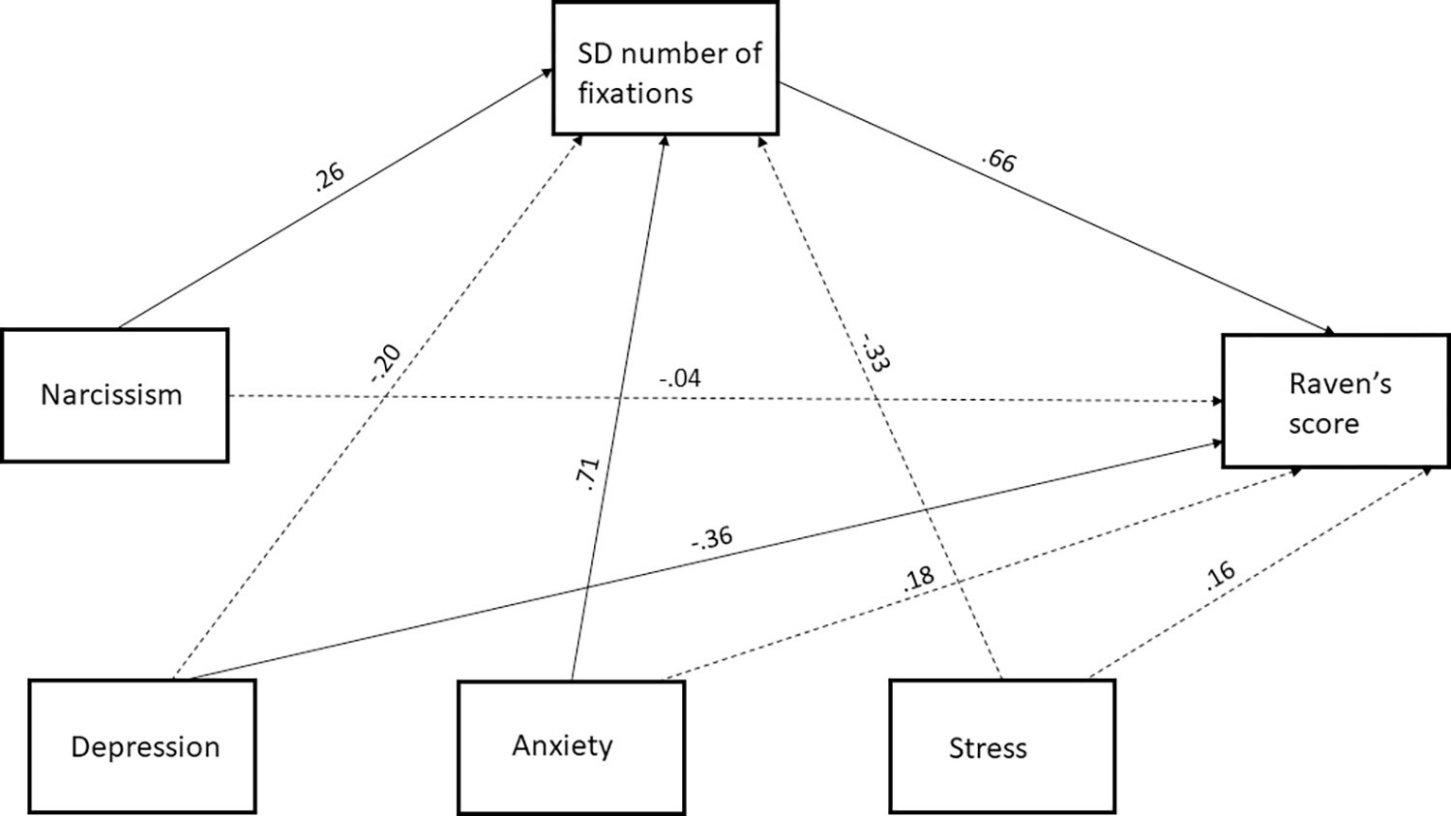
Standardized regression coefficients showing the mediation effects of SD number of fixations in the relation between narcissism and Raven’s score. Solid lines represent significant paths and dotted lines represent non-significant paths.

A representation of the mediation model that we tested is shown in Fig 1. Consistent with our predictions, the findings of the mediation analysis showed no significant direct effect of narcissism on Raven’s score, *B =* -.01, *SE =* .02, *95% CI* [-.05, .03], *t*(49) = -.38, *p =* .70. Α marginal effect of narcissism on SD number of fixations was found, *B =* 5.65, *SE =* 2.83, *95% CI =* [-.04, 11.35], *t*(49) = 1.99, *p =* .05, such that higher narcissism scores were associated with a higher SD number of fixations. SD number of fixations was found to have a significant effect on Raven’s score, *B* = .01, *SE* = .001, *95% CI =* [.004, .008], *t(*49) = 5.84, *p* < .001, such that a higher SD number of fixations was associated with a better performance at the Raven’s test. This finding provided support for hypothesis one.

A significant indirect effect of narcissism on Raven’s score mediated by SD number of fixations was found, *B =* .03, *SE* = .02, *95% CI* = [.0026, .0750]. This significant mediation suggests that participants who had higher narcissism scores displayed a greater variation in the number of fixations across trials, which in turn was associated with higher Raven’s score. This finding provided support for hypothesis four.

Regarding the co-variates included in the model, a significant effect of anxiety on SD number of fixations was found, *B* = 14.26, *SE* = 4.00, *95% CI =* [6.22, 22.30], *t(*49) = 3.56, *p* < .001, such that higher anxiety scores were associated with a higher SD number of fixations. Depression had a significant effect on Raven’s score, *B* = -.05, *SE* = .02, *95% CI =* [-.1, -.01], *t(*49) = -2.34, *p* = .02, such that higher depression scores were associated with a poorer performance at the Raven’s test.

### Narcissism as a predictor of visual attention measures

The findings of the multivariate regression analyses showed a marginal positive association between narcissism and SD number of fixations, *F(*1, 49) = 3.98, *p =* .05. These findings suggest that participants, who scored higher on narcissism, displayed a greater variation in the number of fixations and number of saccades across trials. Regarding the co-variates, the findings showed that higher anxiety significantly predicted a higher overall number of fixations, *F(*1, 49) = 10.7, *p =* .002, higher SD number of fixations, *F(*1, 49) = 12.7, *p =* .001, higher overall number of saccades, *F(*1, 49) = 10.7, *p =* .002, higher SD number of saccades, *F(*1, 49) = 12.7, *p =* .001 and a lower average saccade amplitude, *F(*1, 49) = 4.55, *p =* .04. Higher stress significantly predicted a higher SD of fixation duration, *F(*1, 49) = 5.14, *p =* .03. This finding provided support for hypothesis two.

### Visual attention measures as predictors of Raven’s score

The findings of the linear regression showed that SD number of saccades, *B* = .005, *t(*45) = 2.39, *p =* .02, average fixation duration, *B* = .001, *t(*45) = 2.8, *p =* .007, and average saccade duration, *B* = .002, *t(*45) = 3.61, *p =* .001, positively predicted Raven’ score. The overall model explained 7.9% of the variance and significantly predicted Raven’s score, *F(*8, 45) = 9.25, *p <* .001. These findings suggest that visual attention measures are significantly associated with Raven’s score and provide support for hypothesis three. Correlations between the visual attention measures are presented in supplementary material III in S1 File.

## Discussion

This pre-registered, quasi-experimental study explored associations among grandiose narcissism, visual attention and cognitive performance under stress. Previous research [19] provided evidence of a link between narcissism and improved cognitive performance in stressful situations. The current explorational work aimed to provide insight into the potential cognitive mechanisms that may underlie this association. We were expecting that for participants that were highly stressed variation in number of fixations will be low contributing to poorer attention distribution at task, which in turn could lead to lower performance. Because we had evidence to suggest that narcissism predicts better responses under stress, we hypothesised that individuals scoring high on grandiose narcissism will exhibit better attention distribution as measured by variation in number of fixations achieving higher scores on the Raven’s test.

While grandiose narcissism did not directly predict Raven’s scores, it predicted them indirectly positively through better (more flexible) visual attention distribution. These findings suggest that participants who scored higher on grandiose narcissism displayed more variability in the number of eye fixations launched across trials, which in turn led to higher performance. This suggests that they employed more flexibility in adapting their cognitive strategies while completing the task, which aligns with the findings of previous research which demonstrated that strategy flexibility is linked to improved performance in problem-solving tasks [44].

Interestingly, participants who scored higher on narcissism showed more item-dependent variability in solving the task, which suggests that they were more successful at adapting their problem-solving strategy to meet the demands of the task, while under stress. This greater variability in eye fixation behaviour may potentially be interpreted as evidence for the use of improved adaptive cognitive strategies as reflected in higher Raven’s scores for individuals that scored higher on grandiose narcissism. This effect was not observed either for the other two Dark Triad traits, Machiavellianism and psychopathy, or for healthy self-esteem (see supplementary material III in S1 File). A possible interpretation of this effect is that the inflated sense of self-worth, typical for grandiose narcissists, may contribute to ignoring stressful stimuli (e.g., negative feedback) using their cognitive resources (e.g., visual attention) to perform well at a given cognitive task. Together the results corroborate recent evidence from another experimental study that showed that individuals who scored higher on grandiose narcissism performed better at a mathematical task under stress [19]. Furthermore, they highlight the well-reported association between grandiose narcissism and fewer symptoms of psychopathology. For example, through its positive relationship with mental toughness, grandiose narcissism has been shown to associate with lower scores on depression and stress in three independent sample [6,7]; and directly to fewer behavioral difficulties in adolescence [8]. Results from extant research indicate that individuals who score higher on grandiose narcissism may be highly goal oriented, respond proactively to stressors, and exhibit better mental health outcomes.

These findings also support the previously reported link between visual attention and cognitive performance through the use of adaptive strategies. For example, it was previously demonstrated that the use of holistic strategies while performing a mental rotation task is associated with fewer eye fixations and saccades and that, in turn, the use of holistic strategies is linked to improved performance in the task [55]. As mentioned above, individuals that score higher on grandiose narcissism may be better able to adapt their strategy while performing complex problem-solving tasks under stress, which links to higher performance in the task [44]. The current study builds upon the findings of past research through demonstrating that the strategies employed during problem-solving tasks may depend on personality and individual differences, and that these adaptive strategies could be detected using a novel eye-tracking paradigm.

The findings of the current work should be interpreted in the light of several limitations. Firstly, the self-report data on personality may be influenced by common-method variance and social desirability, particularly in the context of the assessment of dark traits. Secondly, we only assessed grandiose narcissism using a measure that assesses dark traits unidimensionally. Previously, an Exploratory Graph Analysis of Dark Triad domains showed eight separate facets of narcissism [3] and future research could benefit from an exploration of how these different facets of narcissism impact cognitive performance under stress. Thirdly, additional experimental conditions with varying degrees of stress exposure could be included in this paradigm. The present study aimed to have two separate conditions, “low stress” and “high stress”. However, the two different conditions did not yield any significant differences in terms of experienced stress by the participants hence they were merged into one still stressful condition. It would be valuable to assess whether the indirect impact of grandiose narcissism on cognitive performance becomes weaker or stronger depending on differential levels of stress exposure.

Another important point to consider is whether the variability in eye fixation behaviour displayed by participants with higher narcissism scores could be interpreted as strategy flexibility or if there is an alternative explanation for it. Previous research has demonstrated that variability in cognitive test scores could be attributed to mental noise, e.g. neuroticism [56,57]. If so, then the findings of the current work could potentially suggest a link between narcissism and neuroticism as an indicator of arousal, which leads to an increase in performance. This interpretation may be plausible, as shown by the positive correlation between anxiety and SD number of fixations discovered in the SEM analysis (Fig 1). It could suggest that anxiety as an arousal factor may yield a positive effect on performance, specifically for individuals who display higher narcissism scores. This may suggest that narcissists not only display resilience against the negative, psychopathological symptoms of anxiety [7], but they may also benefit from the increased levels of arousal.

Finally, the sample size was perhaps the biggest limitation of this work. Future research should implement this paradigm with a bigger and more representative participant sample in order to enhance the generalisability of the findings. This is important because psychology undergraduate student samples tend to score lower on self-report measures of dark traits. As such, it would be interesting to replicate the findings using samples with means and standard deviations on Dark Triad traits that are closer to those observed in the general population.

In conclusion, this study provided novel evidence for the indirect positive effect of grandiose narcissism on cognitive performance. Grandiose narcissism was found to predict higher Raven’s scores through better visual attention distribution in a stressful situation. Of particular interest is the fact that, this effect was only observed for grandiose narcissism as compared to the other two dark traits and healthy self-esteem. As such, the present study provides additional evidence to support the view that personality traits, specifically dark traits, cannot be simply perceived as being positive or negative. Instead, it is essential that we study their adaptive versus their maladaptive aspects across various contexts. Typically, personality research demonstrates reliable associations between traits and important outcomes such as performance. However, the mechanisms through which a “non-cognitive factor” may impact on cognitive performance remain underexplored. From a theoretical perspective, the current work proposes a possible mechanism to explain the previously reported link between narcissism and improved cognitive performance under stress [19]. Our study is novel in that it provides a possible explanation for the link between narcissism and cognitive performance, such that grandiose narcissists may distribute their attention more efficiently under stress and as a result, perform better. Future studies with larger samples should explore deeper the proposed association in order to shed light on which aspects of narcissism are truly beneficial in terms of performance under stress; and the exact mechanisms through which personality contributes to performance and other important life outcomes.

In this endeavour, our study highlights the usefulness of adapting a multi-method approach with eye-tracking to be a promising methodology, when studying personality-intelligence associations. This approach may have the potential to uncover the cognitive mechanisms underlying associations between personality, intelligence and cognitive performance, especially performance when it matters the most, that is, when under stress.

## Supporting information

S1 File Supplementary material I: Description of measures. Supplementary material II: Correlations. Supplementary material III: Additional analyses for Machiavellianism, Psychopathy and Self-esteem.
(DOCX)
