# Supplementary material for: Grandiose narcissism associates with higher cognitive performance under stress through more efficient attention distribution: An eye-tracking study
Source: PLoS One. 2024 May 3;19(5):e0302644. doi: 10.1371/journal.pone.0302644 (PMC11068195; doi:10.1371/journal.pone.0302644)
Supplement: S1 File — Supplementary material II: Correlations. Supplementary material III: Additional analyses for Machiavellianism, Psychopathy and Self-esteem. (DOCX) [file pone.0302644.s001.docx]

**Supplementary Materials**

**Supplementary material I. Description of measures**

***Narcissism, Psychopathy and Machiavellianism*** were measured using the The Short Dark Triad questionnaire (SD3; Jones & Paulhus, 2013) that assesses the Dark Triad of personalities, narcissism (*M* = 2.58, *SD* = .55, α = .65), psychopathy (*M* = 1.87, *SD* = .56, α = .75) and Machiavellianism (*M* = 2.9, *SD* = .49, α = .64). The SD3 has 27 items, 9 for each scale, which are measured on the 5-point scale where 1 is ‘Strongly disagree’ and 5 is ‘Strongly agree’.

***Depression, Anxiety and Stress*** were measured using the Depression, Anxiety and Stress scale (DASS21; Lovibond & Lovibond, 1995). This is a 21-item measure of symptoms of depression (*M* = 1.74, *SD* = .77, α = .89), anxiety (*M* = 1.86, *SD* = .6, α = .75) and stress (*M* = 2.14, *SD* = .66, α = .8) and responses were given on the 5-point scale where 1 is ‘Strongly disagree’ and 5 is ‘Strongly agree’.

***General cognitive ability*** was measured using Raven’s Progressive Matrices (RPM; Raven et al., 1998), which consists of five sets (A to E) of 12 items each, 60 items overall, presented in order of increasing difficulty. Sets A and B consist of 6 response options, sets C, D and E consist of 8 response options. To calculate overall IQ score, correct responses were dummy coded as ‘1’ and incorrect responses were coded as ‘0’. The mean of correct and incorrect dummy coded responses was taken as a measure to indicate the overall performance at the task (IQ score).

***Perceived stress pre- and post-task*** was measured using the Acute Stress Appraisals scale (ASA, Mendes et al., 2007). This scale consists of a pre-task version that captures appraisals of the stressor prior to the action/performance of the task (*M* = -1.12, *SD* = 1.44) and a post-task questionnaire that assesses individuals’ perceptions of the demands and resources after the task (*M* = .50, *SD* = 1.51). Responses are given on the 7-point scale where 1 is ‘Strongly disagree’ and 7 is ‘Strongly agree’. In order to calculate pre- and post-task stress, the mean of the resource items was subtracted from the mean of the demand items.

***Self-esteem*** was measured using the Rosenberg Self-esteem scale (Rosenberg, 1965). The scale consists of 10 items that assess global self-worth by measuring positive and negative feelings about the self. Responses are given on the 4-point scale where 1 is ‘Strongly disagree’ and 4 is ‘Strongly agree’ (*M* = 2.87, *SD* = .40, α = .84).

**Supplementary material II. Correlations**

**Table 4. Correlations between visual attention measures. Significant correlations at p < .001 are highlighted in bold.**

| Fixation Fixation Saccade Saccade Saccade RT Blink Blink Fixation Fixation Saccade Saccade Saccade RT Blink Blink  count duration count amplitude duration mean sum duration count duration count amplitude duration SD count duration  sum mean sum mean mean mean SD SD SD SD SD SD SD |
| --- |
| Fixation  count sum  Fixation .18  duration sum  Saccade **1**  .18  count sum  Saccade -.37 -.30 -.36  amplitude  mean  Saccade -.15 **-.48** -.14 **.66**  duration  mean  RT mean **.96** .33 **.96** -.28 -.03  Blink sum .35 -.23 .36 .27  **.60**  .42  Blink -.10 -.42 -.09 .39 **.82** .02 .34  duration  mean  Fixation **.90** .07 **.90** -.27 .07 **.89 .47** .09  count SD  Fixation -.04 .41 -.04 -.05 .03 .09 -.05 .03 .04  duration SD  Saccade **.90** .07 **.90** -.27 .07 .**89**  **.47**  .09 1 .03  count SD  Saccade -.31 -.29 -.30 **.90 .70** -.22 .36 .37 -.19 .15 -.19  amplitude  SD  Saccade -.15 -.16 -.15 .15 .35 -.08 .18 **.55** -.08 .18 -.08 .25  duration SD  RT SD **.85** .18 **.85** -.24 .15 .**92** .45 .22 **.95** .07 **.95** .18 -.04  Blink sum SD .44 -.20 .44 .13 **.53 .50 .89** .40 **.59** -.13 **.59** .21 .18 **.60**  Blink -.12 -.26 -.11 .21  **.53** -.02 .19 .**81**  -.02 .05 -.02 .25 **.88** .09 .26  duration SD |
|  |

**Supplementary material III. Additional analyses for Machiavellianism, Psychopathy and Self-esteem**

The findings of the linear regressions were similar to the ones for narcissism. Specifically, Machiavellianism did not directly predict Raven’s score, *B* = .04, *t*(49) = 1.37, *p =* .18. However, the overall model (including co-variates) explained 4.6% of the variance and significantly predicted Raven’s score, *F*(4, 49) = 3.28, *p =* .02; higher depression scores were associated with significantly lower Raven’s score, *B* = -.07, *t(*49) = 2.41, *p =* .02; and higher anxiety scores were associated with significantly higher Raven’s score, *B* = .11, *t(*49) = 3.04, *p =* .004.

Psychopathy did not directly predict Raven’s score, *B* < .001, *t*(49) = 0.02, *p =* .99. The overall model explained 4.3% of the variance and significantly predicted Raven’s score, *F*(4, 49) = 2.71, *p =* .04; higher depression scores were associated with significantly lower Raven’s score, *B* = -.07, *t(*49) = 2.46, *p =* .02; higher anxiety scores were associated with significantly Raven’s score, *B* = .11, *t(*49) = 3.03, *p =* .004.

Self-esteem did not directly predict Raven’s score, *B* = .01, *t*(49) = 0.15, *p* = .88. The overall model explained 4.3% of the variance and significantly predicted Raven’s score, *F*(4, 49) = 2.72, *p* = .04; higher depression scores were associated with significantly lower Raven’s score, *B* = -.07, *t*(49) = 2.42, *p* = .02; and higher anxiety scores were associated with significantly higher Raven’s score, *B* = .11, *t*(49) = 2.91, *p* = .005.

The findings of the multivariate regression analyses showed that, unlike narcissism, there was no significant associations for Machiavellianism, psychopathy or self-esteem with any of the visual attention measures assessed in this study. Likewise, the findings of the mediation analyses showed no significant direct effects of Machiavellianism, psychopathy and self-esteem on Raven’s score and no significant indirect effects of Machiavellianism, psychopathy and self-esteem on Raven’s score as mediated by SD number of fixations.

These findings show that only narcissism (of the other two dark traits and self-esteem) associated directly with better distribution of visual attention; and indirectly with higher Raven’s score.
